# High Capacity and High Efficiency Maple Tree-Biomass-Derived Hard Carbon as an Anode Material for Sodium-Ion Batteries

**DOI:** 10.3390/ma11081294

**Published:** 2018-07-26

**Authors:** Yuesheng Wang, Zimin Feng, Wen Zhu, Vincent Gariépy, Catherine Gagnon, Manon Provencher, Dharminder Laul, René Veillette, Michel L. Trudeau, Abdelbast Guerfi, Karim Zaghib

**Affiliations:** Center of Excellence in Transportation Electrification and Energy Storage, Hydro-Québec, 1806 Boulevard Lionel-Boulet, Varennes, QC J3X1S1, Canada; Wang.Yuesheng@ireq.ca (Y.W.); Feng.Zimin@ireq.ca (Z.F.); Zhu.Wen@ireq.ca (W.Z.); Gariepy.Vincent@ireq.ca (V.G.); Gagnon.Catherine3@ireq.ca (C.G.); Provencher.Manon@ireq.ca (M.P.); Laul.Dharminder@ireq.ca (D.L.); Veillette.Rene2@ireq.ca (R.V.); Trudeau.Michel@ireq.ca (M.L.T.); Guerfi.Abdelbast@ireq.ca (A.G.)

**Keywords:** hard carbon, sodium ion batteries, high capacity, high coulombic efficiency, NaTFSI

## Abstract

Sodium-ion batteries (SIBs) are in the spotlight because of their potential use in large-scale energy storage devices due to the abundance and low cost of sodium-based materials. There are many SIB cathode materials under investigation but only a few candidate materials such as carbon, oxides and alloys were proposed as anodes. Among these anode materials, hard carbon shows promising performances with low operating potential and relatively high specific capacity. Unfortunately, its low initial coulombic efficiency and high cost limit its commercial applications. In this study, low-cost maple tree-biomass-derived hard carbon is tested as the anode for sodium-ion batteries. The capacity of hard carbon prepared at 1400 °C (HC-1400) reaches 337 mAh/g at 0.1 C. The initial coulombic efficiency is up to 88.03% in Sodium trifluoromethanesulfonimide (NaTFSI)/Ethylene carbonate (EC): Diethyl carbonate (DEC) electrolyte. The capacity was maintained at 92.3% after 100 cycles at 0.5 C rates. The in situ X-ray diffraction (XRD) analysis showed that no peak shift occurred during charge/discharge, supporting a finding of no sodium ion intercalates in the nano-graphite layer. Its low cost, high capacity and high coulombic efficiency indicate that hard carbon is a promising anode material for sodium-ion batteries.

## 1. Introduction 

The room-temperature sodium-ion battery was proposed as an alternative candidate for large-scale energy storage due to sodium’s abundance in the Earth′s crust and its low cost [1,2,3]. In addition, aluminum foil can be used in place of copper as the anode current collector to further decrease the cost because sodium does not alloy with aluminum [4]. Up to present, numerous cathode materials have been evaluated, such as the P2 or O3-layered oxide Na_x_MO_2_ (0.5 < *x* < 1, M:Ni, Co, Mn, Fe, Cr, Ti, etc.) [5,6,7,8,9,10,11,12,13,14,15,16,17,18], tunnel-type Na_0.44_MnO_2_ [19,20], Na_0.66_[Mn_0.66_Ti_0.34_]O_2_ [21], Na superionic conductor (NASICON)-Na_3_V_2_(PO_4_)_3_/C [22], ferrocyanide-like copper hexacyanoferrate (CuHCF) [23], nickel and copper hexacyanoferrate (Ni-CuHCF) [24], etc. In contrast, the choices of anode materials for sodium-ion batteries are limited to carbonaceous materials, alloys, Ti-based oxides and some organic compounds. Although alloy materials such as Sb/C [25,26], Sn/C [27], SnSb/C [28] and P [29,30] can deliver a high specific capacity, the destruction of their structure by large volume expansion during reaction with Na leads to cycling instability. Ti-based oxides that include Na_2_Ti_3_O_7_ [3,31], O3-layered NaTiO_2_ [32], spinel-Li_4_Ti_5_O_12_ [33] and P2-layered Na_0.66_[Li_0.22_Ti_0.78_]O_2_ [34] exhibit a suitable sodium storage voltage and stable cycling, but their low electronic conductivity, low capacity, and low coulombic efficiency or multiple-phase mechanism hinders their applications. Organic compounds, such as Na_4_C_8_H_2_O_6_ [35] and Na_2_C_8_H_4_O_4_ [36], have been studied as the anode for SIBs and found to deliver a large reversible capacity with good capacity retention. Nevertheless, suffering from poor electronic conductivity and low initial coulombic efficiency makes them less promising for commercial development. Among the available anode candidates for SIBs, carbonaceous materials [37] hold the most promise considering the various sources of precursors (such as sugars [38], apple [39], banana [40], cotton [41], peanut shells [42], etc.) and cost. However, their reversible capacity is less than 300 mAh/g and initial columbic efficiency is less than 80%. Therefore, improving the capacity and initial columbic efficiency are major steps to satisfying the requirements for hard carbon’s practical applications.

In this study, we report the electrochemical performance of hard carbon derived from a precursor of maple trees branches as anodes for sodium-ion batteries. By heating the precursor at 1400 °C for 6 h, the hard carbon obtained exhibited approximately 332 mAh/g reversible capacity and 88.03% initial coulombic efficiency at a 0.1 C rate with NaTFSI/EC:DEC. At the one-hundredth cycle, 92.3% capacity was observed at a 0.5 C rate. These benefits, along with their low cost, make hard carbons a promising anode material for sodium-ion batteries.

## 2. Results and Discussion 

### 2.1. Structure and Morphology

**Figure 1:**
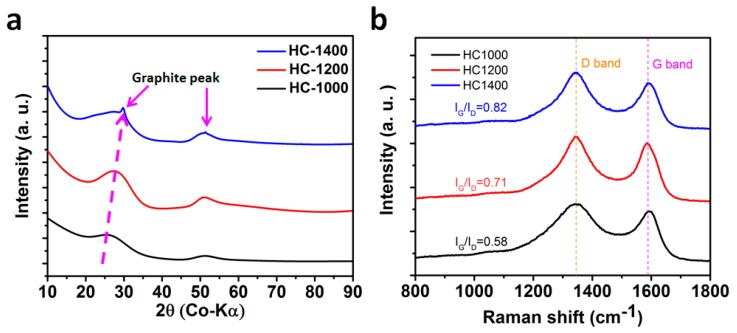
Structure analysis of hard carbon (HC) obtained under different conditions. (**a**) X-ray diffraction (XRD) patterns and (**b**) Raman spectra normalized by the peak height of the G band of HC carbonized at temperatures of 1000 °C, 1200 °C, and 1400 °C.

The X-ray diffraction (XRD) spectra of the structure of hard carbon formed at different pyrolysis temperatures are shown in Figure 1a. Two broad peaks, centered at approximately 29° and 51° are observed in the XRD (Co-Kα radiation) spectra of each of the three samples, which are close to the (002) and (101) planes of graphite (Inorganic Crystal Structure Database ICSD:617290, space group (S.G).:P6_3_/mmc) respectively. The broad peaks suggest the amorphous or nano-crystalline structure of these hard carbon materials. As the heating temperature rose from 1000 to 1400 °C (samples will be named with their corresponding temperature hereafter), the position of the (002) peak shifts to the higher two theta direction, indicating the decrease of the interplanar space between (002) planes due to the structural rearrangement from disorder to short-range order towards the thermodynamically stable state. The XRD spectrum of hard carbon (HC)-1400 shows graphite peaks around 30° and 51°, indicating the co-existence of nano/amorphous-graphite particles. The (101) peak becomes sharper with increased pyrolysis temperature. Raman spectra present the carbon D-band peak at 1340 cm^−1^ (the defect-induced band) and the G-band peak at 1595 cm^−1^ (the crystalline graphite band). The ratio of the intensity of the D-Raman peak and G-Raman peak (I_G_/I_D_) is often used for the characterization of the disorder degree of carbon materials. Figure 1b shows the Raman curves of hard carbons obtained at different temperatures. Interestingly, with increasing pyrolysis temperature, the intensity of the D band increases, but the full width at half maximum (FWHM) gradually decreases, and the value of I_G_/I_D_ keeps increasing from 0.58 to 0.82, which is attributed to the change in the structure from amorphous to planar graphite. The results are consistent with the XRD results. 

**Figure 2:**
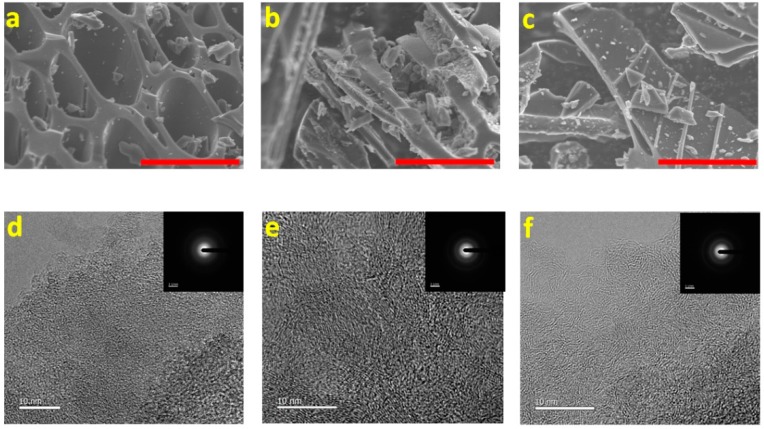
Morphologies of HC obtained at different temperatures observed by scanning electron microscope (SEM) (scale bar: 10 µm): (**a**) HC-1000; (**b**) HC-1200; and (**c**) HC-1400. TEM images and SAED patterns of (**d**) HC-1000; (**e**) HC-1000; and (**f**) HC-1400.

The morphologies of hard carbon at the different temperatures are shown in Figure 2a–c and Figure S1. The size of particles ranges from 1 to 20 μm and the distributions are non-uniform. HC-1000 has a cellulose porous structure, inherited from the tree branch, with some small particles, whereas HC-1400 contains large platy particles greater than 10 µm. The high-resolution transmission electron microscopy (HRTEM) images of hard carbon at the different temperatures are displayed in Figure 2d−f (inset with selected area electron diffraction (SAED)). There is no obvious long-range ordered structure observed in the transmission electron microscopy (TEM) micrographs despite the local ordered structures, representing the nano-graphite domains, becoming more distinct in XRD spectra at higher carbonization temperatures. All SAED patterns exhibit dispersive diffraction rings, which further confirm the disordered microstructure. The diffraction rings become less diffused with increasing carbonization temperature, with some bright spots for HC-1200 and HC-1400, which demonstrates a tendency toward an ordered structure. In addition, some graphitic domains arise in the local ordered structure of HC-1400. Thus, the results of TEM and SAED further demonstrate that the temperature can promote graphitization and improve ordered-ness, which is consistent with the XRD and Raman results.

### 2.2. Sodium Storage Performance in Sodium Ion Batteries

**Figure 3:**
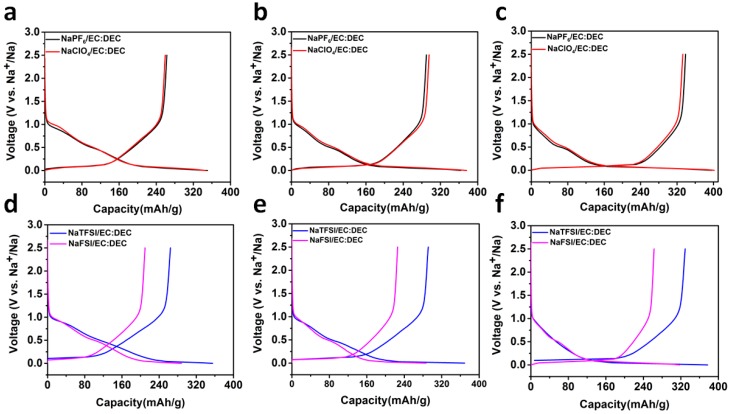
Storage performance of half-cells in different electrolytes. Discharge/charge curves with NaPF6/EC:DEC and NaClO4/EC:DEC electrolytes: (**a**) HC-1000; (**b**) HC-1200; and (**c**) HC-1400. Discharge/charge curves with NaTFSI/EC:DEC and NaFSI/EC:DEC electrolytes: (**d**) HC-1000; (**e**) HC-1200; and (**f**) HC-1400.

**Figure 4:**
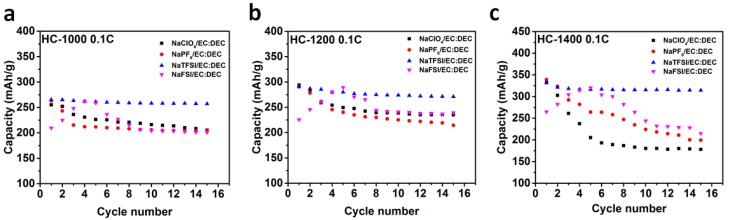
Cycle performance. Cycle performance of HC samples at a current rate of 0.1 C with different electrolytes: (**a**) HC-1000; (**b**) HC-1200; and (**c**) HC-1400.

**Figure 5:**
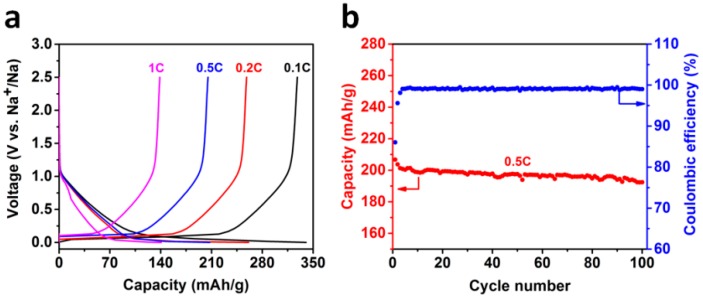
Rate and cycle performance. (**a**) Discharge/charge curves at the different rate from 0.1–1 C; and (**b**) cycle performance of HC-1400 with NaTFSI/EC:DEC at a current rate of 0.5 C.

The low initial coulombic efficiency caused by the formation of a solid electrolyte interphase (SEI) layer limits the application of hard carbon due to the irreversible loss of Na in the cathode. To solve the problem of low initial coulombic efficiency and to form a stable SEI, we optimized the electrolyte by using various sodium salts, such as sodium hexafluorophosphate (NaPF_6_), sodium perchlorate (NaClO_4_), NaTFSI and sodium (I) bis(fluorosulfonyl)imide (NaFSI), which are normally used to improve the low initial coulombic efficiency and cycle performance of the electrode materials in sodium ion batteries. The electrochemical properties of anodes which were derived from hard carbons, processed at different temperatures, as anode materials were tested in coin cells using sodium metal as the counter electrode. Figure 3a–f and Table 1 shows the initial discharge/charge curves and summary of initial coulombic efficiency in 1mol NaPF_6_, NaClO_4_, NaTFSI and NaFSI in EC:DEC. The voltage range is 0 to 2.5 V. The first galvanostatic discharge/charge curves at a current rate of 0.1 C (30 mA g^−1^) are shown in Figure 3a–f. The voltage profiles of all electrodes exhibit two distinct regions: (1) A gradual voltage decay around 0.15 1.2 V and (2) a plateau around 0.15 V. The results obtained in 1M NaPF_6_/EC:DEC electrolyte show that HC-1000 displays a relatively low charge capacity (262 mAh/g) and a low coulombic efficiency of 75.1%, while the HC-1400 electrode exhibits a high charge capacity of 337 mAh/g and a coulombic efficiency of 86.1%. The cells in NaClO_4_, NaTFSI and NaFSI in EC:DEC electrolytes yield similar results, which are attributed to the low brunauer-emmett-teller (BET) surface areas (Table S1), and the capacity percentage of the plateau region of around 70%. We also examined the influence of different salts on the capacity of electrodes. Using NaPF_6_, NaClO_4_ and NaTFSI as salts, the initial coulombic efficiency and capacity of the cells with the same electrode are comparable or better than those using NaFSI. Figure 4 illustrates the cycle performance of HC electrodes in different electrolytes. The capacity fades rapidly for the conventional NaPF_6_ and NaClO_4_ electrolytes. In the case of the NaFSI electrode, a low capacity is obtained in the first cycle; the reversible capacity increases after five cycles and then decreases rapidly. However, the NaTFSI electrolyte exhibits stable capacity with around 95% capacity retained after few cycles at 0.1 C rate. Note that after optimization, HC-1400 outperforms the existing carbon anode materials for sodium-ion batteries. The rate capability of HC-1400 in 1M NaTFSI/EC:DEC electrolyte was tested in a sodium ion cell, and the results in Figure 5a show that the reversible capacities are 332, 264, 210 and 145 mAh/g at constant current rates of C/10, C/5, C/2 and 1 C, respectively. The HC-1400 electrode exhibits capacity retention of 92.3% at a current rate of 0.5 C after 100 cycles. The coulombic efficiency after the third cycle is nearly 99.5%. 

### 2.3. Structure Evolution

**Figure 6:**
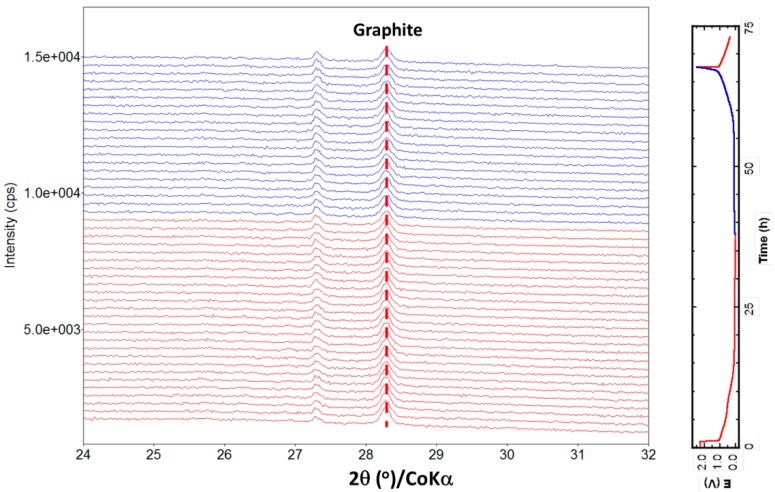
Structure evolution during electrochemical cycle. In situ XRD patterns collected during the first discharge/charge of the HC-1400 electrode cycled between 0–2.5 V at 0.05 C rate with 1M NaTFSI/EC:DEC electrolyte.

The sodium storage mechanism was investigated by in situ XRD. Until now, three different Na storage environments and three corresponding models have been reported in the literature for Na interactions with hard carbon: (i) Adsorption on the surface’s active sites; (ii) nano pore filling analogous to adsorption; and (iii) intercalation between the graphene layers with suitable distance—spacing. The electrochemical results show two distinct voltage regions: A slope above 0.15 V and a plateau below 0.15 V. A number of studies were conducted aiming at elucidate Na interactions with hard carbon, but the Na storage mechanisms in different voltage regions are still being debated. In 2001, Stevens and Dahn [43] first reported Na storage in glucose-pyrolyzed hard carbon, and assigned the voltage sloping region to Na-ion insertion in the graphene layers and the low voltage plateau region to Na filling/plating into nanopores. Later, Komaba et al. [44]. corroborated this mechanism by spectrometric characterization. In their study, ex situ XRD showed that the (002) peak of hard carbon shifted from 2θ = 23.4° to 21–22° when the voltage is reduced to 0.1 V, then returned to the pristine position when charged back to 2.0 V, indicating the reversible Na removal from graphene sheets. Recently, Tarascon and co-workers [45] investigated the structural variation of polyacrylonitrile-derived carbon nanofibers. They concluded that the slope region between 1.0 and 0.1 V is due to Na-ion adsorption on disordered isolated graphene sheets, and the 0.1 V plateau is due to mesopore filling by Na ions. Our results are more consistent with those of Tarascon et al. For HC-1400, the graphite peak around 29° becomes obvious. In situ XRD is employed to directly investigate whether the sodium ions intercalate in the nano graphite sheet. Figure 6 shows the (002) diffraction peak evolution in XRD patterns of the HC-1400 electrode cycled at 0.05 C rates in the voltage range of 0 to 2.5 V during the initial discharge and charge. No obvious shift of the graphite peak was observed when the cell discharged to the cut-off voltage, implying that neither the high potential slope nor the low voltage plateau can be assigned to sodium intercalation between graphitic layers.

## 3. Methods

### 3.1. Materials Synthesis

The hard carbon samples were produced from direct pyrolysis of inner parts of a maple tree. Firstly, pieces from the inner of the maple tree have been treated in 1M HCl for 2h, washed three times using deionized water and dried in the oven at 100 °C for 6 h. Secondly, pieces from the inner of the maple tree were carbonized for 6 h in a tube furnace under argon flow. The pyrolysis temperatures were 1000 °C, 1200 °C and 1400 °C, respectively, and the obtained product was ground in a mortar for use. The prepared samples that were obtained are designated as HC-1000, HC-1200 and HC-1400, respectively.

### 3.2. Characterization 

The carbonized structure was characterized by an X-ray diffractometer (SmartlabRigaku, (Rigaku Corporation: Tokyo, Japan) using Co−Kα radiation (1.788965 Å). The sample morphologies were investigated with a scanning electron microscope (TESCAN) (TESCAN Corporation: Libušina tř. 21623 00 Brno—Kohoutovice Czech Republic). Transmission electron microscope images (TEM) and selected area electron diffraction (SAED) patterns were obtained using a cold- Field Emission Gun (FEG) TEM-Hitachi HF3300 (Hitachi high technologies: Tokyo, Japan) operated at 300 kV.

### 3.3. Electrochemistry

Composite electrodes were fabricated from samples of HC-1000, HC-1200, or HC-1400, and Super P and polytetrafluoroethylene (PVDF) binder mixture in a mass ratio of 80:12:8 was coated onto Al foil. The loading of electrodes for HC-1000, HC-1200 and HC-1400 are 3.85 mg/cm^2^, 3.67 mg/cm^2^ and 4.15 mg/cm^2^, respectively. The electrolyte consists of 1M NaPF_6_ (>98%, Sigma-Aldrich, Oakville, ON, Canada), NaClO_4_ (>99%, Sigma-Aldrich, Canada), sodium trifluoromethanesulfonimide (NaTFSI, >99%, Solvionic, Toulouse cede, France) or sodium bis(fluorosulfonyl)imide (NaFSI, >99%, Solvionic, Toulouse cedex, France) in ethylene carbonate (EC) and diethyl carbonate (DEC) (3:7 in volume). A sodium foil was used as the counter electrode and glass fiber (GF/D 47, Whatman, UK) was used as the separator. All the operations were conducted in an argon-filled glovebox. The discharge and charge tests were conducted on EC-Lab battery test system (Bio-Logic acquired Uniscan Ltd., Vaucanson, France) in a voltage range of 0–2 V at various C-rates at room temperature. 

## 4. Conclusions

In summary, we have developed a new hard carbon derived from maple trees. With an optimised electrolyte such as the NaTFSI/EC:DEC, initial coulombic efficiency is up to 88.03% and the specific capacity is 332 mAh/g. Furthermore, 92.3% of the capacity is still retained after 100 cycles at 0.5 C. Most importantly, we observed no graphite peak shift in in situ XRD, indicating no sodium intercalation to graphite. We believe these findings not only provide a novel anode material for sodium-ion batteries, but also expand the electrolyte options to improve initial coulombic efficiency or capacity for the commercialization of sodium-ion batteries.

## Figures and Tables

**Table 1:** Initial efficiency of hard carbon as anode with different electrolytes.

Electrode Electrolyte	HC-1000 1st Charge/Discharge (ICE)	HC-1200 1st Charge/Discharge (ICE)	HC-1400 1st Charge/Discharge (ICE)
**NaPF_6_/EC:DEC**	262/349 (75.1%)	290/362 (80.1%)	337/391 (86.1%)
**NaClO_4_/EC:DEC**	260/347(74.8%)	296/376(78.5%)	332/401(82.7%)
**NaTFSI/EC:DEC**	264/354(74.5%)	291/368(79.1%)	332/378(88.03%)
**NaFSI/EC:DEC**	209/287(72.8%)	225/286(78.6%)	264/317(83.1%)

Note: ICE is initial coulombic efficiency.

## Supplementary Materials

The following are available online at http://www.mdpi.com/1996-1944/11/8/1294/s1, Figure S1: SEM images of HC obtained at different temperatures (a) HC-1000, (b) HC-1200 and (c) HC-1400 observed by SEM (scale bar:50 µm), Table S1: BET parameters. BET surface area for HC-1000, HC-1200,HC-1400.
