# Supplementary material for: High Capacity and High Efficiency Maple Tree-Biomass-Derived Hard Carbon as an Anode Material for Sodium-Ion Batteries
Source: Materials (Basel). 2018 Jul 26;11(8):1294. doi: 10.3390/ma11081294 (PMC6117673; doi:10.3390/ma11081294)

Supplementary Materials to:

High Capacity and High Efficiency Maple Tree-Biomass-Derived Hard Carbon as an Anode Material for Sodium-ion Batteries

Yuesheng Wang, Zimin Feng, Wen Zhu, Vincent Gariépy, Catherine Gagnon, Manon Provencher, Dharminder Laul, René Veillette, Michel L. Trudeau, Abdelbast Guerfi and Karim Zaghib *

Center of Excellence in Transportation Electrification and Energy Storage, Hydro-Québec, 1806 boulevard Lionel-boulet, Varennes, QC. J3X1S1, Canada; Wang.Yuesheng@ireq.ca (Y.W.); Feng.Zimin@ireq.ca (Z.F.); Zhu.Wen@ireq.ca (W.Z.); Gariepy.Vincent@ireq.ca (V.G.); Gagnon.Catherine3@ireq.ca (C.G.); Provencher.Manon@ireq.ca (M.P.); Laul.Dharminder@ireq.ca (D.L.); Veillette.Rene2@ireq.ca (R.V.); Trudeau.Michel@ireq.ca (M.L.T.); Guerfi.Abdelbast@ireq.ca (A.G.)

* Correspondence: Zaghib.Karim@ireq.ca

Supplementary Tables

Supplementary Table S1 | BET parameters. **BET surface area** for HC-1000, HC-1200,HC-1400.

Sample	Surface Area (m^2/g)<br>QuadraSorb (Quantachrome Instruments)
HC-1000°C	72.7
HC-1200°C	5.5
HC-1400°C	6.4
Standard<br>Surface area: 98.17±7.53 m^2/g	97.5

Supplementary Figures

Supplementary Figure S1 | SEM images of HC obtained at different temperatures (a) HC-1000, (b) HC-1200 and (c) HC-1400 observed by SEM (scale bar:50 μm).

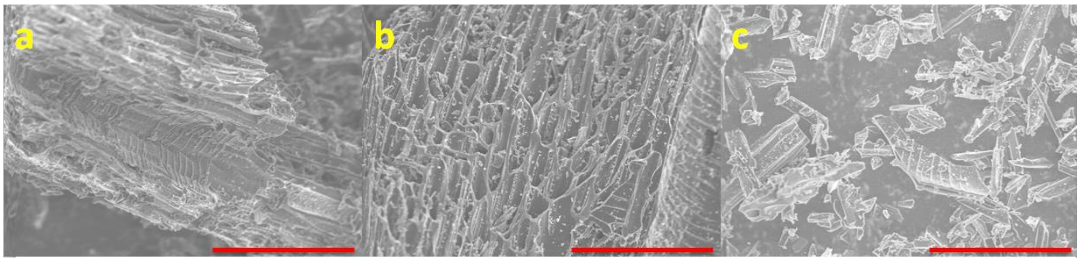

Supplement: Supplementary file 1 [file materials-11-01294-s001.pdf]
